# Advancements in Tissue Engineering: A Review of Bioprinting Techniques, Scaffolds, and Bioinks

**DOI:** 10.1177/11795972241288099

**Published:** 2024-10-01

**Authors:** Shervin Zoghi

**Affiliations:** School of Medicine, University of California, Davis, Sacramento, CA, USA

**Keywords:** Bioprinting, 3D printing, tissue engineering, bioink, scaffold

## Abstract

Tissue engineering is a multidisciplinary field that uses biomaterials to restore tissue function and assist with drug development. Over the last decade, the fabrication of three-dimensional (3D) multifunctional scaffolds has become commonplace in tissue engineering and regenerative medicine. Thanks to the development of 3D bioprinting technologies, these scaffolds more accurately recapitulate in vivo conditions and provide the support structure necessary for microenvironments conducive to cell growth and function. The purpose of this review is to provide a background on the leading 3D bioprinting methods and bioink selections for tissue engineering applications, with a specific focus on the growing field of developing multifunctional bioinks and possible future applications.

## Introduction

The origins of 3D bioprinting began in 1986, when Chuck Hull patented stereolithography, a novel 3D printing process for rapidly creating models in a layer-by-layer fashion.^1^ He soon realized that this process could apply to other materials as well, and in the years since, rapid improvements in the underlying technology have enabled 3D printing to grow at an astronomical 26.2% annually and become the primary modality for customized prints across many industries.^2^ Despite this growth, the adoption of 3D printing in the healthcare industry has not progressed as rapidly, in large part due to the technological and regulatory challenges of the medical field. While 3D printing has previously been utilized for the construction of patient-specific anatomical models for pre-operative planning^3,4^ and low-cost prosthetics^5,6^, 3D bioprinting has only recently emerged as a promising candidate for a major paradigm shift in tissue engineering and the study of tissue models.^7-10^

Tissue engineering is a multi-disciplinary field that uses biomaterials to restore damaged tissue function and assist with drug development.^11^ While traditional techniques use petri dishes for 2-dimensional modeling of tissues, additive manufacturing has made it possible to more accurately recapitulate in-vivo conditions by fabricating 3-dimensional models to better mimic the spatial and chemical attributes of native tissues.^11^ For example, researchers and commercial entities have used 3d manufacturing to study drug metabolism using actual human hepatocyte models and patient derived xenografts,^12^ with the goal of developing personalized drugs and therapeutics. This technology has also enabled scientists to precisely mimic the in-vivo microenvironment of tumors outside of the human body and provide a faster pipeline to test chemotherapies on human cell lines rather than on animal cells.^13^

3D bioprinting also ushers in the possibility for fabricating and transplanting autologous tissue analogs. According to the United Network for Organ Sharing, there are over 105 000 people currently on a waiting list to receive an organ. Many of these patients will die before they are able to receive a new organ, while those that do receive them will be on lifelong immunosuppressive medications that places them at an increased risk for illness and malignancies.^14,15^ By being able to print and seed organs with the patient’s own cells, the risk of rejection that is associated with donor transplantation drops significantly.^16^ Although scientists have been able to successfully engineer several basic types of tissues and organs for small scale clinical trials, research toward the development of functional vascular organs through scaffold and bioink advancements are still ongoing.^17^

While many unique possibilities exist for revolutionizing the medical field, this review aims to streamline much of the literature to focus on the leading developments in 3D bioprinting from the past decade and the forthcoming challenges hindering their widespread adoption. Specifically, the purpose of this study is to evaluate the leading bioprinting techniques, explore the role and benefits of various scaffolds and bioinks used in 3D bioprinting, and outline the future direction and challenges facing this technology.

## 3D Bioprinting Methods

The primary technologies used to fabricate 3-dimensional tissues are droplet, extrusion, and laser-based printing. Depending on the bioinks used, costs, and desired properties such as cell viability, print speeds, and resolution, different considerations should be made as to which printing modality should be selected. The pros and cons of each of the three major classes of 3D bioprinters are discussed below.

### Extrusion-based printing

Extrusion-based 3D bioprinters are the most commonly utilized bioprinters because of their simplicity, affordability, and consistency during printing. They can be classified in one of two categories: either pneumatically driven using compressed air to drive a syringe and nozzle, or mechanically driven by a motor or linear piston.^18,19^ Extrusion printers are ubiquitous in printing plastics, particularly with materials such as PLA (polylactic acid) and ABS (acrylonitrile butadiene styrene), as well as a number of other industries.^20^ As a result, bioengineering researchers have also adopted the technology and are able to use much of the same software and hardware to print a range of biologic structures including organoids, valves, hepatic tissues, ears, and more.

Depending on the material’s viscosity, extrusion-based bioprinters generally use either compressed air or mechanical screw to extrude the material.^21-23^ Compared to inkjet-based and laser-based printers, extrusion-based printers are capable of printing higher cell densities and more viscous materials.^24,25^ While higher viscosity materials provide superior structural support for scaffolds, lower viscosity materials are better for biological processes.^26,27^ The reason for this is that although extrusion printers can print a wide range of materials, during the printing process, the cells are subjected to additional mechanical forces that are proportional to the pressure, viscosity, shape, and speed of the printed part. This is because the pressurized bioinks move through a nozzle with a fixed geometry, which causes the cells to experience different velocity gradients based on their radial position.^28^ Near the nozzle walls, the cells experience the maximum velocity gradient and shear stress, which is the result of material slippage along a plane parallel to the direction of stress.^29^ This is the primary cause of cell deformation and apoptosis in extrusion-based printing.^30-32^

One of the newer bioprinting techniques that aims to improve cell viability and fabricate shelf-ready cell-laden constructs involves extrusion-based cryobioprinting.^33^ Scientists were recently able to modify an extrusion-based printer to print scaffolds directly onto a cooled −40°C surface.^34^ This gradient allows directional freezing and phase separation that results in multizone scaffolds, thereby better mimicking the complex structure of native cartilage.^35^ While cryobioprinting is still in its infancy, preliminary studies have demonstrated improvements in the ability of these complex scaffolds to serve as platforms for mesenchymal stem cell attachment and survival.

With certain considerations such as using shear-thinning fluids, modifying the bioink formulations, and slowing print speeds, the damage during extrusion-based printing can be significantly reduced. Furthermore, since the majority of newer, commercially available extrusion-based bioprinters can accommodate multiple printers for each of the different bioinks or supportive materials, the printer setup and settings can be further optimized to improve the structural integrity of the printed tissue.^36^ Understanding the many relevant factors that go into printing the various components of tissue, such as print speed, material selection, extrusion temperature, nozzle diameter, and fluid viscosity, thus empowers researchers to carefully select and optimize the quality of the printed constructs for drug development and tissue transplantation.

### Droplet-based printing

Unlike extrusion-based bioprinting, droplet-based bioprinting is non-contact and reproduces patterns onto a substrate using tiny ink droplets that are generated primarily by either thermal, piezoelectric, or electromagnetic actuators.^37^ These actuators can selectively control the flow of droplets with resolutions between 20 and 100 microns.^38^ While the precision and accuracy are significantly advantageous, droplet-based bioprinting has a much slower manufacturing time than extrusion-based bioprinting.

Due to its simplicity and reproducibility, droplet-based bioprinting is also highly versatile and widely used in small-scale studies involving regenerative medicine, pharmaceutical development, and cancer research.^39^ Although capable of also printing hydrogels, cells, and biologic substances, droplet printing is far more limited in how viscous of materials it can print because of the minimal forces that can be achieved during printing.^39^ Higher viscosity substrates can thus lead to uneven deposition and nozzle obstructions, as well as shear stress induced apoptosis due to the small printhead orifice.^40^ As a result, droplet-based prints thus have reduced structural integrity, increased size constraints stemming from a lack of immediate vascularization, and restrictions due to porosity constraints.^39^ Despite these common limitations, because newer droplet printers use microfluidic nozzles, research has started to show that prints are improving in fidelity and integrity by better preparing monodispersed viscous microspheres using the phase inversion method.^41^

### Laser-assisted printing and digital light projection printing

Laser-assisted printing is a technique initially developed to deposit metals onto an optically transparent substrate.^42^ Then, in 2000, Odde and Renn and Ren modified this technique to print embryonic chick spinal cords, showing that this process can be used to construct arrays that are hundreds of cells with micrometer-scale precision.^43^ Modern laser-assisted bioprinting techniques rely on laser pulses to deposit cells from a ribbon/donor slide onto the receiver substrate underneath in a pre-specified pattern.^44^ The donor slide is generally composed of a layer of glass, metal, and bioink—when the laser contacts the metal, it absorbs the energy and induces the hydrogel to vaporize and releases the newly detached cell.^43^ Of the bioprinting techniques discussed, laser-assisted bioprinting generally has the greatest yield (cell viability >95%) and resolutions of up to one cell.^45,46^ This is a result of the ability to finely tune many of the print characteristics, such as laser energy, surface tension, wettability, air gap between the substrate and donor slide, and bioink viscosity.^38^

Despite these advantages, laser-assisted bioprinting is severely limited in terms of productivity, usability, and speed.^44^ Even with the cell suspensions being exposed to 100% relative humidity during printing to counteract cellular dehydration, the printing process can only continue for about 10 minutes before encountering issues due to the movement of the slides during processing.^47-49^ Furthermore, having a homogeneous distribution of cells increases their susceptibility to gravity-induced changes to the cell layer, thus occasionally requiring supplementation with 10% glycerol in sodium alginate.^47,50^ While these are unique challenges to laser-assisted bioprinting, it is still a useful tool for researchers to use in order to model and better understand the proliferation and interactions of cells in well-controlled environments.

Although similar to laser-assisted printing, digital light projection (DLP) bioprinting projects light from a UV source onto a liquid resin to cure it into a series of square 3D pixels.^51^ As a result, the resolution of the print is defined by a singular pixel, which enables newer DLP bioprinters to print significantly higher fidelity models (<20 microns) at faster fabrication speeds relative to most comparable laser-assisted bioprinters.^52^ Furthermore, unlike laser-assisted printing which uses a fixed-intensity laser beam, DLP printing is more customizable and allows for variability in the intensity of UV light for various resins. This customizability has helped lead to innovative new approaches for fabricating various microtissue models in the field, such as in tumor organoid and tumor-on-chips modeling.^52^ While the DLP systems are optically capable of fabricating basic microvascular models, extensive developments in light absorbing bioinks are needed before complex vasculature and multi-material DLP bioprinters are ubiquitous.^53^

## Key Scaffold Properties

One of the key components of 3D bioprinting is the development of a multi-functional scaffold. This supportive structure is integral in facilitating the adhesion and proliferation of new cells, as well as determining the characteristics and functionality of the final tissue.^54^ As the scaffold encounters the culture media, the protein adsorption mediates adhesion and integrin release, thereby facilitating the actions of cellular signaling, extracellular matrix (ECM) deposition, and cell proliferation and differentiation.^55^ Thus, the success of cell attachment and viability depends on the intrinsic and extrinsic scaffold material properties, the cell types being grown, and the growth media provided. Deciding which scaffold materials to use and what post-processing methods to pursue therefore has significant implications for the success of developing models that can accurately mimic native tissues in order to better promote integration and transplantation. Some of the scaffold properties that determine the functionality of the tissue models explored below include surface hydrophobicity, conductivity, vascularization, pore size, and surface roughness.

One of the most well-studied and easily measurable properties of a biomaterial is its hydrophobicity. This can be measured by evaluating the contact angle formed between a water droplet and the solid material surface—a contact angle of greater than 90° means the solid is hydrophobic, while less than 90° means hydrophilic. Studies have shown that hydrophobic surfaces tend to better absorb proteins while hydrophilic surfaces are able to better resist protein absorption.^56^ Furthermore, recent studies have shown that many cell types, such as neuronal cells and osteoblast-like cells, exhibit rapid proliferation and differentiation on hydrophilic surfaces.^55^ Additionally, these surfaces have also been shown to discourage the growth of contaminants such as bacteria or viruses.^57^

Another important property is scaffold conductivity. Conductive polymers are able to deliver electrical stimulation, which improves proliferation and differentiation for some cells such as nerve cells.^58^ This is especially important for numerous fields within regenerative medicine, such as Cardiac Tissue Engineering. By some estimates, the indirect and direct cost of heart disease will reach almost half a trillion dollars by 2030.^59^ While traditional medications, implantable devices, and heart transplants are important and lifesaving, they have a number of drawbacks including side effects, additional costs, and limited supply.^60^ Cardiac Tissue Engineering subsequently emerged as a possible future strategy to regenerate or replace damaged tissues. Due to the complex mix of muscular and conductive tissues of the heart, synthetic cellular scaffolds needs to be able to mimic the native cardiac extracellular matrix.^61^ Thus, a key feature of engineering cardiac and neuronal tissue is ensuring optimum conductivity for electron transmission, biocompatibility, and electrical stimulation of the cell culture.^62^

The primary challenge facing tissue engineering is ensuring adequate vascularization in order to support growth, function, and viability of tissues requiring blood supply.^63^ This process is facilitated by the sprouting of new capillaries using angiogenic factors, called angiogenesis.^64^ While current technology makes it possible to generate small-scale cartilage, bone, and skin,^65-67^ larger and more metabolically active organs like livers, hearts, and kidneys require more comprehensive vascular networks that have a nutrient supply within 300 microns of all cells.^68,69^ These networks are crucial to effectively distribute oxygen and nutrients, as well as remove waste products, from the tissues.^63^

One key feature to facilitating neovascularization of a scaffold and tissue ingrowth is the scaffold pore size—while the optimal size varies with application,^70^ a balance needs to be struck between the pores being large enough to allow for cell penetration into the scaffold bulk, while also being small enough to optimize cell retention and surface area.^63^ Several studies have recently been conducted to ascertain the optimum pore size for the various tissue engineering applications, showing the ideal size is as follows: skin regeneration is 20 to 150 microns,^71^ bone regeneration is 100 to 400 microns,^72,73^ and hepatocytes and fibroblasts are approximately 20 microns.^70^ However, a study by Van Tienen et. al. specified that vascularized fibrous tissue should be at least 30 microns in diameter in order to allow for simultaneous tissue invasion.^74^

Depending on the scaffold material that’s selected, it may not be immediately usable for certain applications based on its intrinsic properties. For instance, one of the most common scaffolds utilized is a polylactic acid (PLA) due to its biodegradability and minimal impact to the environment. While cost-effective and ubiquitous, it lacks the appropriate adhesiveness to be effective for adequate cellular proliferation. Thus, significant efforts have gone into developing post-processing techniques that can address some of the intrinsic property weaknesses. One way this has been achieved is by incorporating surface modification techniques like Plasma Discharge to increase surface roughness. Studies have shown that in many cell types, such as human fetal osteoblasts and mesenchymal stem cells, increased surface roughness corresponds to improved cellular adhesion and proliferation.^75,76^

Scaffolds clearly play an integral role in the success of bioprinting tissues. They need to be able to support, and even induce, a cascade of events leading to tissue repair and generation.^77^ Furthermore, they need to be reproducible and cost-effective, critical components of achieving regulatory approval and widespread adoption in drug development and tissue transplantation. By understanding the role of scaffolds and the different properties of the various fabrication technique and materials, researchers can better select or develop suitable scaffolds for their unique applications.

## Bioinks

Consisting of a mix of living cells and biomaterials, bioinks help ensure a supportive environment during and after the production of cell and tissue models.^7^ Studying the properties, namely mapping the rheological behavior of the bioinks such as the viscosity at different shear stresses, is an important step toward understanding and optimizing their printability and reproducibility.^78^ By measuring the viscosities across low, medium, and high shear rates (representing the bioink at rest ( $∼0.1\;s^{-1}$ *)*, flowing through the nozzle *( $∼100\;s^{-1}$ )*, and actively extruding *( $∼1000\;s^{-1}$ )*, respectively), researchers can better characterize the bioink’s shear-thinning behavior.^78^ Furthermore, due to the limited number of rapid manufacturing options commercially available in bioprinting, it’s essential to identify the optimal materials that are compatible with the existing printing methods and scaffolding. As a result, a myriad of biomaterials has been developed in response to this, including, but not limited to, hydrogels, polymers, ceramics, decellularized extracellular matrix (dECM), and biomolecules (Tables 1–3).

**Table 1.** Common materials used for 3D bioprinting.

	Hydrogels	Polymers	Ceramics	dECM	Biomolecules
**Material**	• Collagen • Gelatin • Alginate • Hyaluronic acid • Chitosan • Fibrin • Silk • Cellulose	• Polycaprolactone • Polylactic acid • Polylactic-co-glycolic acid • Poly-L-lactic acid	• Hydroxyapatite • Tricalcium phosphates	• Skin • Bone • Cartilage	• Growth Factors • Messenger RNA • Micro RNA

**Table 2.** Advantages and disadvantages of the different classes of biomaterials.

	Hydrogels	Polymers	Ceramics	dECM	Biomolecules
**Pros**	• High water content resembling natural tissues • Supports angiogenesis, cell adhesion, and growth • Easily tailorable for various applications	• Controlled degradation for regeneration • Low cost • Wide ranging properties • Easily reproducible during fabrication	• High mechanical strength and stiffness • Osteoconductive • High bioactivity • Mimics mineralized bone tissue	• Minimal immune response • Reproduce nearly all requirements of ECM	• Enhanced tissue regeneration and healing • Precise control over many biological pathways
**Cons**	• Poor mechanical strength, limiting load-bearing capability • Rapid degradation • Low printing fidelity	• Bio-compatibility concerns, can induce inflammation • Does not mimic natural tissue environment	• Hard and brittle • Difficulty achieving complex structures • Limited biodegradability	• Difficulty with standardization and preservation • Limited mechanical strength	• Chemically unstable outside controlled environments • Distribution challenges

**Table 3.** Brief overview of the recent work on common biomaterials used for 3D bioprinting.

Biomaterials	Cells/biomolecules	Select novelties	REF
**Collagen** **Collagen/GelMA**	Cardiomyocytes HUVECs MSCs VEGF Chondrocytes	Bioprinting of functional tissue architectures; vascularization; controlled degradation	Stratesteffen et al^79^, Lee et al^80^, Diamantides et al^81^, Wu et al^82^, Rhee et al^83^, Kim et al^84^
**Gelatin/silk fibroin** **Gelatin/alginate** **Gelatin/alginate/graphene oxide** **Gelatin/GelMA** **Gelatin/polyurethane**	hADSCs Immunoregulators MSCs ECs BMSCs	Extrusion-based low-temperature 3D printing; anticancer immunity; tunable mechanical properties and degradation rates	Li et al^85^, Zhang et al^86^, Zhang et al^87^, Ojansivu et al^88^, Hsieh and Hsu^89^, Shao et al^90^, Ouyang et al^91^
**GelMA** **GelMA/Methacrylated silk fibroin** **GelMA/alginate** **GelMA/HA** **GelMA/gelatin** **GelMA/bone particles** **GelMA/PEGDA** **GelMA/ZnO**	BMSCs LSCs MFCs HSFs Fibroblasts hMSCs VEGF	Cell encapsulation and support; suitable printability; structural and biological compatibility; extended degradation time; rapid printing of nerve conduits; light-controlled growth factor release; drug release	Shao et al^90^, Ouyang et al^91^, Gao et al^92^, Osi et al^93^, Murphy and Atala^94^, Jian et al^95^, Bolívar-Monsalve et al^96^, Zhou et al^97^, Chai et al^98^, Zhong et al^99^, Adib et al^100^, Xu et al^101^, Shao et al^102^, Ratheesh et al^103^, Erdem et al^104^, Chimene et al^105^, Tao et al^106^, Ying et al^107^, Van Belleghem et al^108^, Siebert et al^109^, Cidonio et al^110^, Zhang et al^111^
**Alginate** **Alginate/dECM**	HBECs HLSMCs	Bioprinting human tissues; heart model	De Santis et al^112^, Mirdamadi et al^113^
**Chitosan** **Chitosan methacrylate/PCL** **ChMA**		Microfiber leaching, freeze-drying, and superficial active modification; antimicrobials; DLP printing	Du et al^114^, Teoh et al^115^, Shen et al^116^, Zhou et al^117^
**Silk fibroin** **Silk Fibroin/graphene oxide**	Chondrocytes Turbinate-derived MSCs	Photocurable silk fibroin	Kim et al^118^, Hong et al^119^, Kim et al^120^
**PCL** **PCL/PGS** **PCL/alginate/dECM**	MSCs TGF- β3	Elastic 3D-printed hybrid polymeric scaffold; biomimetic; perfusable hierarchical networks	Sun et al^121^, Shafiee et al^122^, Lei et al^123^, Rathan et al^124^, Yang et al^125^
**Hap/gelatin/alginate** **Hap/GelMA** **Hap/alginate/acrylamide**	MSCs Osteoblasts	Two-step crosslinking; biomimetic; multilayer scaffolds	Zhang et al^126^, Wüst et al^127^, Zhou et al^128^, Liu et al^129^
**calcium phosphate/collagen** **β-tricalcium phosphate (β-TCP)/Ag/GO**		Multi-step printing; antibacterial and osteogenic activity	Zhang et al^130^, Inzana et al^131^
**Matrigel/alginate/gelatin**	VEGF	Sustained release profiles of bioactive VEGF	Poldervaart et al^132^

### Polymers

Biodegradable polymers, such as polylactic acid (PLA), poly(lactic-co-glycolic acid) (PLGA), poly(L-lactic acid) (PLLA), polycaprolactone (PCL), and poly(glycerol–sebacate) (PGS), have recently gained traction in tissue engineering. Their appeal stems from their enhanced biocompatibility, degradability, cost-effectiveness, and ease of processing.^133,134^ However, a notable drawback involves the production of acidic byproducts during degradation, requiring precise control to maintain a safe micro-environment^135^ Another drawback is that polymers such as PCL and PLGA require high working temperatures, which is not compatible with cells without modifications such as combining with ceramics, nanoparticles, and hydrogels to significantly enhanced scaffold performance for tissue engineering applications.^121-123,136^

Among these biodegradable polymers, PCL is more widely utilized in 3D bioprinting.^121-123,136^ With a melting temperature of about 60°C, PCL can be converted into filaments for extrusion-based printing, typically at temperatures ranging from 100 to 180 °C. However, its slow degradation, owing to its hydrophobic nature, limits its suitability for shorter-term applications, favoring scenarios involving long-term implantation spanning several years. Yet, PCL as a scaffold material poses challenges for proper cell growth and differentiation, leading to research exploring composite materials to enhance its properties.^121-123,136^ For example, Sun et al designed 3D-printed constructs with a gradient structure releasing dual factors, specifically tailored for cartilage regeneration.^121^ This involved melting PCL to create the gradient-supporting structure while bioprinting mesenchymal stem cells (MSC) laden hydrogel into the microchannels between PCL fibers from distinct syringes.

Moreover, Shafiee et al engineered biomimetic medical-grade PCL dressings with pore architecture and anisotropic mechanical characteristics through 3D printing.^122^ These dressings, produced via electrowriting, were seeded with human gingival tissue multipotent MSCs and preserved using a clinically approved method. Application of these printed PCL dressings led to reduced wound contracture and significantly improved skin regeneration, as demonstrated in a rat model over a 6-week period, offering promising outcomes for enhancing skin wound healing while minimizing scarring.^122^

#### Hydrogels

Consisting of 3D polymer networks with high water retention capacity, hydrogels are essential in tissue engineering for simulating native extracellular matrix (ECM) environments, promoting angiogenesis, cell adhesion, and growth. Their biocompatibility and tunable properties like stiffness, porosity, and degradation rates make them ideal for creating custom tissues and organs. Facilitated by their non-Newtonian nature, Hydrogels can encapsulate and distribute multiple cell types and bioactive molecules in intricate structures.

During production, cells are encapsulated by mixing a cell suspension with a pre-cursor solution, followed by crosslinking or polymerizing the mixture. Further crosslinking can be achieved using stimuli like temperature, light, or ion concentration. Innovations such as hybrid hydrogels and bioactive molecule incorporation allow precise control over mechanical strength, degradation, and bioactivity. However, toxic cross-linking chemicals and uncontrolled degradation pose safety concerns, prompting research into composite materials to address these limitations.

Collagen, the most abundant structural protein in animal and human tissues, is advantageous for tissue engineering due to its structural integrity and support.^79-84^ It is biocompatible, biodegradable, porous, and capable of enhancing cell growth and adhesion.^80^ Collagen scaffolds can also be cross-linked or modified with other materials to address the slow gelation and low mechanical strength.^79,80^ Techniques like using methacrylated gelatin with collagen or rapid pH changes for collagen self-assembly enhance the capability of bioprinting complex structures.^79^ Incorporating bioactive components like growth factors and cells also enhances collagen’s regenerative potential, though continued research is needed in order to ensure optimum print fidelity and vascularization.

Gelatin, derived from collagen hydrolysis, exhibits qualities like non-cytotoxicity, water solubility, biocompatibility, biodegradability, and minimal immunogenicity.^137^ Often used in the form of GelMA, gelatin has excellent bioprinting capabilities.^90-108^ Innovations like GelMA and methylacrylate-modified chitosan (ChMA) bioinks allow precise control over rheological behavior and mechanical strength, enhancing scaffold properties.^93^ Unique bioprinting systems and cryoprotective bioinks further expand the potential applications of gelatin in tissue engineering.^138,139^

Alginate, a biocompatible and cost-effective polysaccharide, is widely used in inkjet and direct ink writing (DIW) printing.^94,96,112^ Although alginate exhibits lower cell adhesion compared to other natural polymers, blending with other polymers or incorporating dECM enhances its properties significantly in supporting tissue maturation and angiogenesis.^96,112^

Hyaluronic acid (HA) plays an integral role in the ECM, supporting cell proliferation and migration.^97,140,141^ HA’s customizable degradation rate, adjusted through variations in crosslinking and molecular weight, makes it ideal for scaffold use in 3D bioprinting.^97,140,141^ Techniques like integrating photocuring enhance scaffold properties for cartilage regeneration.^140^ Molecular cleavage approaches combining HA with GelMA further improves the mechanical properties and fidelity of bioprinted tissues.^141^

Chitosan, a biodegradable polymer, boasts exceptional biocompatibility and anti-bacterial properties.^114^ Its ability to form porous structures makes it suitable for cell transplantation and tissue regeneration.^115^ Innovations like hemostatic chitosan sponges with interconnective microchannels demonstrate superior pro-coagulant and hemostatic properties, promising for clinical translation.^114^

Silk fibroin (SF) is favored for its exceptional biocompatibility, mechanical stability, non-toxicity, and minimal bacterial adherence.^118,142^ SF-based bioinks require blending with other polymers and optimizing rheology but show significant potential for applications like cartilage reconstruction using advanced 3D bioprinting techniques.^85^

### Ceramics

Bioactive ceramics, particularly hydroxyapatite (HAP) and tricalcium phosphate (TCP), have emerged as prominent materials in tissue engineering, especially for bone regeneration applications.^93,126,138^ These materials are useful due to their chemical similarity to bone minerals and their capacity to bond with both soft and hard tissues, offering natural and osteo-inductive surfaces conducive to bone tissue development.^130^ However, their inherent brittleness and high melting temperatures (>1000℃) pose challenges for 3D bioprinting applications, necessitating the development of composite materials that blend ceramics with bioink matrix materials such as hydrogels or polymers.^130,143^ The bioactive ceramic materials can thus provide mechanical strength to the hydrogel scaffolds, while also releasing bioactive ions promoting angiogenesis and osteogenesis as they degrade.^144^

HAP, comprising approximately 70% of bone tissue structure, exhibits intrinsic osteoconductive and bioactive properties that promote tissue growth. Despite these advantages, HAP’s limitations, including poor mechanical properties and slow degradation rates, have prompted researchers to explore composite materials that combine HAP with hydrogels and polymers to enhance mechanical and functional attributes.^93,126,138^ Recent advancements in this field have yielded promising results, demonstrating the potential of ceramic-based composites in addressing complex challenges in tissue regeneration and disease modeling.

Osi et al. developed a printable hydrogel ink by combining GelMA, methylacry-late-modified chitosan (ChMA), and nanohydroxyapatite (nano-HAP). This innovative blend exhibited minimal adverse effects on mechanical rigidity and cytocompatibility, while demonstrating shear-thinning properties and exceptional structural integrity. Zhang et al created a 3D-printed gradient hydrogel scaffold mimicking osteochondral tissue structure, incorporating varying concentrations of nano-HAP in different layers. This approach yielded superior repair and regeneration outcomes when enriched with bone marrow stromal cells, as evidenced by comprehensive physicochemical, mechanical, and biological evaluations.^126^

Furthermore, Wu et al employed an extrusion-based bioprinting method to establish an in-vitro model for multiple myeloma, featuring a bone marrow-like microenvironment. This advanced model, comprising an outer mineral-containing sheath and an inner soft hydrogel core, demonstrated the ability to sustain patient-derived MM cells for up to 7 days, significantly outperforming conventional 2D cell cultures.^138^

By addressing the limitations of traditional ceramic materials while leveraging their beneficial properties, researchers are developing increasingly sophisticated and effective solutions for tissue regeneration and disease modeling. The integration of ceramic-based composites with advanced manufacturing techniques, such as 3D bioprinting, holds significant promise for the future of tissue engineering and regenerative medicine.

### Decellularized extracellular matrix (dECM)

Despite efforts by scientists to develop cell-printed structures, the interaction between cells and materials remains a major barrier to mass adoption. The complexities arising from cell-material interactions have prompted researchers to recreate conditions like the natural microenvironment, leading them to incorporate living tissue elements as bioink.^95,112,124^

The ECM serves as a composite framework comprising various components including, but not limited to, collagen, glycosaminoglycans, chondroitin sulfate, and elastin, within the cellular environment.^112^ Decellularized ECM materials are obtained from desired tissues where cells are systematically removed, preserving the ECM structure. Utilizing dECM proves to be an optimal approach as it nearly replicates all aspects of the ECM. Typically, dECM is processed into a powder and dissolved in a cell-friendly solution to create the bioink.^95^ However, bioinks derived from dECM often exhibit poor printability and physical properties, resulting in restricted shape fidelity and scalability.^112^ To address this limitation, researchers have explored blending dECM with other natural polymers for 3D bioprinting.^95,112,124^

Jian et al developed a bioink derived from meniscal ECM, combining GelMA and meniscal ECM, to address printability and cytocompatibility simultaneously for tissue engineering applications.^95^ Rathan et al created a cartilage ECM based alginate bioink for the bioprinting of cartilaginous tissues.^124^ These bioinks are printable, supporting MSC viability post-printing and robust chondrogenesis in-vitro. Furthermore, De Santis et al designed a tissue-specific hybrid bioink comprising alginate reinforced with ECM derived from decellularized tissue.^112^ Their proof-of-concept involved 3D bioprinting human airways using regionally specified primary human airway epithelial progenitor and smooth muscle cells. The resulting airway lumens remained patent with viable cells for 1 month in-vitro, demonstrating evidence of differentiation into mature epithelial cell types found in native human airways.

### Biomolecules

The recent studies in tissue engineering have shown a concerted effort to enhance scaffold properties by incorporating a diverse range of materials such as hydrogels, polymers, and ceramics. These materials play a crucial role in bolstering the mechanical strength, antimicrobial capabilities, and biological performance of scaffolds. By introducing biomolecules into the scaffold structure, a bioresorbable composite is subsequently formed, which further optimizes the ability for tissue regeneration. The controlled breakdown of these scaffolds, with biomolecules uniformly dispersed throughout or concentrated in specific sections, allows for a gradual and regulated release of these bioactive compounds. This is promising and is anticipated to significantly enhance cellular activities, including improved cell growth, proliferation, and seamless integration of the scaffold with the surrounding host tissue. As a result, researchers aim to understand how these bioactive components can be strategically used to optimize the performance and efficacy of tissue-engineered scaffolds, thereby advancing the field toward more effective regenerative therapies.

Growth factors, particularly Transforming Growth Factor-beta (TGF-β) and Vascular Endothelial Growth Factor (VEGF), play crucial roles in various biological pathways.^109,121,124,132^ TGF-β, known for its osteo and chondro-inductive properties, stimulates muscle-derived stem cell differentiation and collagen production.^124^ Incorporating TGF-β into scaffolds has shown significant improvements in cell migration, survival, differentiation, and adhesion. For instance, Rathan et al demonstrated that bioinks containing MSCs and TGF-β supported robust chondrogenesis.^124^ Similarly, Sun et al developed bioprinted constructs with dual factor release and gradient structures, fostering an isotropic cartilage regeneration.^121^ VEGF, a principal regulator of angiogenesis, has also been extensively integrated into scaffold systems to enhance angiogenic properties. Poldervaart et al investigated controlled VEGF release from gelatin microparticles within 3D bioprinted scaffolds, demonstrating successful continuous release over 3 weeks in-vitro.^132^

RNA, including messenger RNA and microRNA, has gained attention as a biomolecule in tissue engineering. Despite its inherent instability, researchers have explored various delivery methods to stabilize these molecules.^145^ Recent studies have shown successful integration of miRNA into 3D bioprinted collagen scaffolds, demonstrating significant augmentation of bone growth and repair of bone defects in both in-vitro and in-vivo assessments using rat models.^145^ These findings highlight the promising potential of RNA-based biomolecules in bone tissue engineering applications.

The incorporation of these biomolecules into tissue engineering scaffolds represents a significant advancement in the field, offering potential for improved tissue regeneration and more effective regenerative therapies.

## Current Challenges and Future Directions

Despite the advances that have been made in the last few decades, numerous challenges remain before 3D bioprinting can be used to develop fully functional tissue specific extracellular matrix based bioinks. Currently, most bioprinted models have limited useability due to their rudimentary structures and limited number of cell types that can be printed at a given time. Fabricating complex and functional tissue models require more robust and tailorable cells and supporting materials, along with the capability to integrate vasculature for nutrient and oxygen supply.^146^

One of the major obstacles facing the printability of biologic materials is a result of the inherent limitations of extrusion-based printing, the most popular technique for printing tissues. Although these 3D bioprinters have historically been limited in how accurately they can print finer structures such as hepatocytes and fibroblasts, the newer generation of bioprinters have been shown to print with accuracies up to 20 microns.^70,147^ Furthermore, as addressed earlier, despite its many benefits, one of the major challenges facing extrusion printing is cell membrane damage due to shear stresses from the extruder nozzles during printing. While limited accommodations can be made to incorporate shear-thinning materials and hydrogel precursors that can be crosslinked after deposition, the different hydrogels still have varying properties and gelation techniques; thus, it’s important to utilize a hydrogel that is as biocompatible and non-cytotoxic as possible.^148,149^

A significant obstacle facing the transplantation of bioprinted tissues is the ability to accurately fabricate the vasculature necessary to provide oxygenated blood to these tissues and organs. Following the traditional in-vivo implantation of cell scaffolding constructs, inflammatory-driven angiogenic growth factors initiate vascularization. However, because vascularization relies on vessel growth, it is limited to less than a micrometer a day, resulting in non-vascularized constructs for an extended period of time.^150,151^ While this is acceptable for thinner or avascular tissues like skin or cartilage, ischemic processes can occur in larger tissues and lead to additional complications.^152-154^ As a result, developing an alternative fabrication method is imperative in order to ensure that the printed tissues become perfused more rapidly, thereby reducing the risk of hypoxic processes compromising cellular viability.^155-158^

One of the potential solutions to this requires in-vitro pre-vascularization, which heavily relies on the self-organizing capacity of endothelial cells and co-cultures to form vascular networks.^159-161^ While fabricating these structures in a precise manner through 3D bioprinting is appealing, a more thorough understanding of the cell sources, bioink compositions, scaffold designs, and bioprinting techniques are needed.^45,94,162,163^ Although there have been developments in printing small-scale vascularized tissue constructs and free-standing structures, fabricating interconnected vascular networks is still in its infancy.^164^ The correct development of these are crucial elements of a functional organ because they not only provide oxygen and nutrients to the cells, but also are key toward regulating signaling molecules and cell growth.^165^

Researchers are pursuing the fabrication of highly complex and functional vascularized tissues through either direct-cell-seeding or post-cell-seeding via either laser, droplet, or extrusion-based printing modalities.^94,166,167^ Creating these vascular constructs requires either 1 of 2 techniques: co-culturing vascularization-promoting cells within the scaffolds to generate the vascular networks, or directly fabricating perfusable blood vessels (typically via extrusion or SLA based printing).^168^ For instance, scientists are working on engineering vascularized neural tissues that more accurately recapitulate the complexity of the nervous system and better model the Blood Brain Barrier; the ability to rapidly print donor-specific neural tissues enable physicians to develop more personalized tools that allow the precise modeling of various medications.^169^

Another new tissue engineering technology is the emergence of 4D bioprinting. Building off the infrastructure of existing 3D bioprinters, this new generation of printers additionally adopts the component of time in order to stimulate processing in-situ and no longer treat printed objects as static, but rather as dynamic objects that can simultaneously undergo external stimuli in real time.^170^ Adoption of this requires both an external stimulus and shape adaptable materials. In recent years, scientists have begun experimenting with various shape memory materials (SMM), including shape memory alloys, polymers, hybrids, ceramics, and gels.^171^ There is a significant opportunity for 4D bioprinting to revolutionize tissue engineering, particularly to develop highly complex and customizable materials that currently require additional, cumbersome post-processing.

Despite the advancement in the field and opportunities ahead, several challenges remain before 4D bioprinting can become ubiquitous like 3D bioprinting. There is a significant need to develop 4D software applications capable of handling various shape-changing mechanisms and materials.^172^ Additionally, ongoing improvements to the existing printing modalities are needed, with a specific focus on developing fully automated, high-resolution printers that are inexpensive and commercially viable.^173^ Lastly, continued research is needed on biopolymers, including improving their viability and speeds during printing, as well as developing the capability for providing numerous functions independently for various biomedical applications.^174^
